# Alternatives to DPA and ethoxyquin for preventing the development of superficial scald in apples: A review

**DOI:** 10.1016/j.fochx.2024.101730

**Published:** 2024-08-11

**Authors:** Konstantinos Papoutsis

**Affiliations:** Department of Plant Breeding, The Swedish University of Agricultural Sciences, SE-230 53, Box 101, Alnarp, Sweden

**Keywords:** Chilling injury, Physiological disorders, Pome fruit, Postharvest losses, Postharvest quality, Storage

## Abstract

Apples are one of most economically important crops worldwide with a production of approximately 96 million tons in 2022. During postharvest storage, apple quality can decline due to the development of physiological disorders. Superficial scald is one of the main physiological disorders that develops in apples during cold storage and results in quality deterioration. Superficial scald is controlled by synthetic antioxidants such as diphenylamine (DPA) and ethoxyquin. Both chemicals have been banned from the EU due to their toxicity. The current review provides an update on superficial scald complicated development mechanism and summarizes studies investigating postharvest treatments as alternatives to DPA and ethoxyquin. Ethylene and oxygen are important factors that trigger the development of superficial scald in apples by regulating various metabolic pathways during cold storage. More studies are required to investigate alternatives to synthetic antioxidants and elucidate the contribution level of the different metabolites to superficial scald development.

## Highlights

• Superficial scald development is a complicated phenomenon
• Various mechanisms involved in the development of superficial scald
• More research is required to provide new insights into superficial scald mechanism
• Treatments control scald by modifying fresh produce physiology
• α-farnesene and conjugated trienols are not the main reason for scald development

## 1 Introduction

Apples are one of the most economically important crops worldwide with a production of approximately 96 million tons in 2022 (FAOSTAT, 2022). To achieve a year-round availability apples can be stored for up to 12 months at low temperatures (<1 °C) (Sabban-Amin et al., 2011). During postharvest storage, apple quality can decline due to the development of physiological disorders. Superficial scald is one of the main physiological disorders that develops during postharvest storage in apples and pears and results in significant postharvest losses (Populin et al., 2023; Torres & Sepulveda, 2023). Superficial scald is a chilling injury disorder that is usually visible when fruits are removed from cold storage. However, after extended storage, it can be visible on fruits even prior to removal to warmer temperatures (Lurie & Watkins, 2012). Some cultivars are more susceptible to superficial scald than others. For instance, ‘Granny Smith’, ‘Red Delicious’, ‘Cortland’, and ‘Law Rome’ seem to be the most susceptible to the development of superficial scald (Gong et al., 2021; Yihui et al., 2018). The susceptibility of some cultivars to superficial scald development may be due to differences in physiology and gene expression of the various apple cultivars as a response to storage conditions.

Superficial scald is mainly controlled by the fruit industry using synthetic antioxidants such as diphenylamine (DPA) and ethoxyquin. However, both chemicals (DPA and ethoxyquin) have been banned from the EU due to their toxicity to humans and animals. Therefore, alternatives to the synthetic antioxidants are urgently needed. The maximum residual limits for both chemicals for apples and pears are 0.05 mg · kg^−1^ (COMMISSION REGULATION (EU) 2018/1515, 2018). To date, studies have noted that DPA may inhibit superficial scald development in apples by decreasing respiration via inhibiting the flow of electrons through the cytochrome path, by reducing conjugated trienes, by reducing ethylene production, and by inhibiting laccase activity (Niu et al., 2018; Whitaker, 2000; Yihui et al., 2018). Ethoxyquin has been noted to have a similar mechanism to DPA (Lurie & Watkins, 2012). However, DPA and ethoxyquin mechanisms need to be further investigated and understood. This will provide important information for the development of environmentally and human-friendly postharvest treatments.

To date, one study published in 2012 reviewed alternative treatments to synthetic antioxidants for the prevention of superficial scald in apples and also discussed the mechanism of superficial scald development focusing on α-farnesene and its oxidative products (Lurie & Watkins, 2012). However, recent -omics studies (transcriptomic and metabolomic) have provided additional information on the mechanism of superficial scald development in apples beyond α-farnesene hypothesis. The current research reviews studies conducted mainly between 2018 and 2023 investigating postharvest treatments as alternatives to DPA and ethoxyquin on the prevention of superficial scald development in apples during postharvest cold storage. The mechanism of action of different postharvest treatments is also discussed. The current study also provides new insights into the mechanisms involved in superficial scald development. This information will be useful for breeders to develop scald resistant cultivars in the future.

## 2 Toxicity of diphenylamine (DPA) and ethoxyquin

**Fig. 1:**
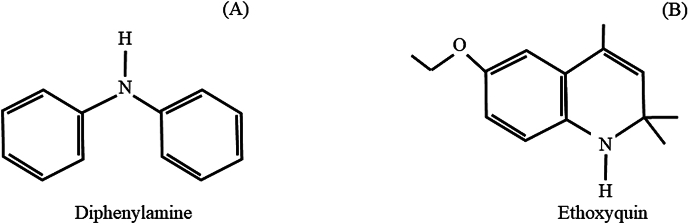
Structure of diphenylamine (A) and ethoxyquin (B).

DPA (C_12_H_11_N) is a chlorinated analogue of aniline and consists of an aromatic amine bound to two phenyl substituents (Fig. 1A) (Ahmed, 2024). DPA is a parent compound of many derivatives which are used for the production of dyes, pharmaceuticals, and photography chemicals (Drzyzga, 2003; Gheni et al., 2023). As previously mentioned, DPA has been extensively used as a synthetic antioxidant for the prevention of the development of superficial scald in apples and pears. However, studies have noted that DPA can be toxic to humans, animals, and the environment (Table 1). Humans can be exposed to DPA through the consumption of fruits and vegetables and during the production and use of DPA through inhalation, skin, and/or eye contact (Ahmed, 2024; Klein, 2014). In humans, DPA may impact the liver, kidneys, blood, and bladder (Ahmed, 2024; Santovito et al., 2012). Liang et al. (2024) noted that newborns can be exposed to DPA through breastfeeding which may pose a risk to their health. Animal studies have noted that DPA exposure can impact the blood, kidneys, liver, and spleen of rats, rabbits, and pigs. For instance, Latif et al. (2023) orally treated pregnant rats with 400 mg DPA·kg^−1^ body weight from the 5^th^ to 19^th^ day of gestation. The authors noted that DPA induced spleen toxicity in pregnant rats and foetuses. Additionally, both mothers and foetuses exposed to DPA developed severe anemia, thrombocytopenia, and leukopenia.

**Table 1:** Evidences on ethoxyquin and diphenylamine (DPA) toxicity in humans, animals, and fish.

**Ethoxyquin**		
	Concentrations of 0.25 and 0.5 mM had *in vitro* cytotoxic effects leading to cell apoptosis or necrosis and damage of genetic material at DNA or chromosome levels.	(Blaszczyk & Skolimowski, 2005)
	Ethoxyquin (5 mg·L^−1^) caused mortality (25%) and deformed bodies or missing eyes (62.5%) in zebrafish. Ethoxyquin decreased the heart rate of the embryos.	(Egloff & Pietsch, 2018)
	Ethoxyquin doses ranging from 1 to 250 μM induced DNA damage in human lymphocytes in a dose-dependent manner.	(Blaszczyk, 2006)
	Zebrafish eggs exposed to 100 μM of ethoxyquin had 95 and 100% mortality after 24 and 48 h, respectively. Ethoxyquin had an effect on larvae development and pigmentation.	(Pradhan et al., 2020)

**DPA**		**References**
	Pregnant rats were orally administrated DPA (400 mg·kg^−1^ body weight). DPA induced spleen toxicity in pregnant rats and foetuses. DPA resulted in severe anemia, thrombocytopenia, and leukopenia in both mothers and foetuses.	(Latif et al., 2023)
	DPA is acutely toxic to rabbits, mice, rats, and many other species. Target organs are blood, kidneys, and liver.	(Klein, 2014)
	DPA treatment showed *in vitro* cytogenetic effects on human cells.	(Santovito et al., 2012)
	DPA has been detected in cow, sheep, goat, and water buffalo milk of animals raised in Italy and France. Mice exposed to 2625 ppm and 5250 ppm of DPA through their diet had darkened spleens and livers.	(Ahmed, 2024)

Ethoxyquin (C_14_H_19_NO) is a clear, viscous, light yellow to dark brown liquid, with an unpleasant odor (Fig. 1B) (Ramis-Ramos, 2003). Ethoxyquin is a cheap product with a very long shelf-life (Ramis-Ramos, 2003). Apart from postharvest applications, ethoxyquin is also used as an insecticide, herbicide, fungicide, plant growth regulator, animal feed additive, and antidegradation agent for rubber (Ramis-Ramos, 2003). The main exposure to ethoxyquin is through the diet of both humans and animals. In humans, ethoxyquin exposure has been shown to cause DNA damage. For instance, Blaszczyk (2006) noted that ethoxyquin doses ranging from 1 to 250 μM induced DNA damage in human lymphocytes in a dose-dependent manner (Table 1). Studies in fish have noted that ethoxyquin exposure can cause physiological and developmental toxicity to aquatic organisms (Table 1) (Egloff & Pietsch, 2018; Pradhan et al., 2020). For instance, Pradhan et al. (2020) noted that zebrafish eggs exposed to 100 μM of ethoxyquin showed 95 and 100% mortality after 24 and 48 h, respectively. Ethoxyquin exposure also had an effect on larvae development and pigmentation. Egloff and Pietsch (2018) noted that ethoxyquin at a concentration of 5 mg·L^−1^ enhanced mortality in zebrafish eggs and caused damage to embryos, such as severe yolk sac deformation and reduced yolk sac transparency. Toxicological information on DPA and ethoxyquin are still limited and more studies are encouraged to investigate the toxic effects of both synthetic antioxidants to human, animal, aquatic life, and the environment.

## 3 Update on superficial scald development

**Fig. 2:**
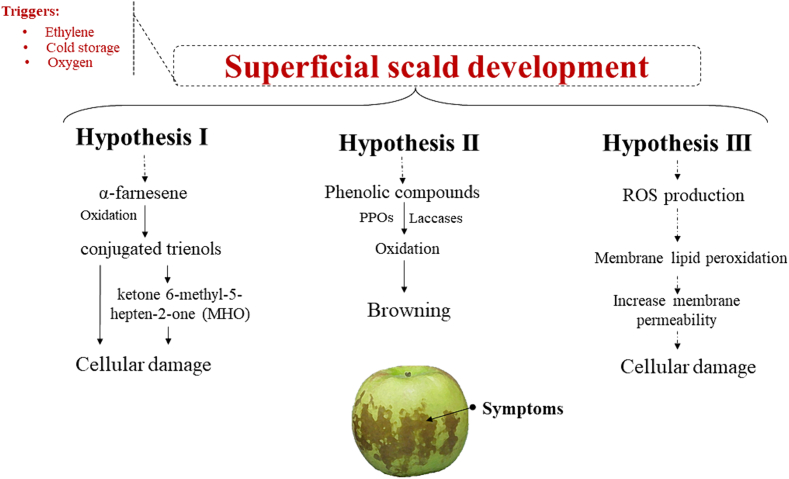
Different hypothesis on superficial scald development.

The most important postharvest parameters that affect scald development include variety, storage temperature, storage duration, and storage atmosphere (i.e., ethylene and oxygen concentration) (Fernández-Cancelo et al., 2022; Populin et al., 2023). Superficial scald is a chilling injury physiological disorder that is usually visible when fruits are removed from cold storage (0–5 °C) and stored at warmer temperatures for a few days. However, after extended storage (>4 months) it can be visible on fruits even prior to removal to warmer temperatures (Lurie & Watkins, 2012). The disorder is characterized by an uneven browning or bronzing of the skin, associated with necrosis of the first 5–6 peel hypodermal cell layers, along with the development of skin wrinkling and pitting with increasing severity (Rudell et al., 2011; Savran & Koyuncu, 2016). Different factors are involved in the development of superficial scald in apples such as low temperatures during cold storage and the presence of ethylene and oxygen in the storage atmosphere (Fig. 2). For instance, cold storage induces the production of superoxide anions which leads to the generation of other reactive oxygen species (ROS) (i.e., OH·, O_2_·, H_2_O_2_) that may result in cell membrane lipid peroxidation that leads to cellular disruption (Devireddy et al., 2021; Fernández-Cancelo et al., 2022; Lu et al., 2014). When apples are transferred to warmer temperatures after cold storage the levels of ROS increase significantly which may contribute to the development of superficial scald symptoms during shelf-life. For instance, Sabban-Amin et al. (2011) noted that a significant increase in ROS levels was recorded in ‘Granny Smith’ apples transferred to shelf-life conditions for 1 week, after 24 weeks of cold storage (0 °C). In addition to ROS production, it has been noted that 6-methyl-5-hepten-2-one (MHO) concentration significantly increases in apples transferred to warmer temperatures after cold storage (Busatto et al., 2018). For many years it has been hypothesized that the main reason for superficial scald development in apples is the oxidation of α-farnesene (sesquiterpene volatile) to conjugated trienols (CTols), which can be converted by a non-enzymatic oxidation into the volatile ketone MHO. Both CTols and MHO can cause cellular damage (Ding et al., 2019). Even though α-farnesene and its oxidation products contribute to the development of superficial scald, recent studies have shown that they are not the main cause for the development of the physiological disorder. The postharvest accumulation of α-farnesene and its oxidation products is closely correlated to ethylene production (Fernández-Cancelo et al., 2022; Karagiannis et al., 2018; Karagiannis et al., 2020; Martins Melo et al., 2021). Ethylene is an important factor that significantly contributes to superficial scald development by regulating various metabolic pathways. Ethylene production modulates the expression of genes involved in α-farnesene biosynthesis such as *hmg2* encoding 3-hydroxy-3-methylglutaryl-CoA reductase (HMGR) which catalyzes the conversion of HMG-CoA to mevalonate and the expression of *MdAFS1* encoding α-farnesene synthase 1, the last enzyme in the α-farnesene biosynthetic pathway (Karagiannis et al., 2018; Rupasinghe et al., 2001). Shelf-life temperatures followed after cold storage have been shown to enhance the activities of enzymes involved in ethylene synthesis such as 1-aminocyclopropane 1-carboxylic acid (ACC) synthase (ACS) and ACC oxidase (ACO) (Fernández-Cancelo et al., 2022), which may partially explain the visibility of superficial scald symptoms after cold storage removal. Additional to ethylene, jasmonic acid a plant hormone associated with plant defense mechanism and signal transduction after wounding, has been shown to contribute to the development of superficial scald by inducing the accumulation of ethylene production (Fernández-Cancelo et al., 2022). Studies have noted that scald development in apples is also related to polyphenol synthesis and oxidation. Among the plethora of phenolics found in apple peels, chlorogenic acid and catechin have been closely correlated with scald development (Busatto et al., 2018; Yihui et al., 2018). Populin et al. (2023) noted higher expression of *phenylalanine ammonia-lyase (PAL)* and *polyphenol oxidase (PPO)* in ‘Granny Smith’ apples during cold storage. PAL is an important enzyme that catalyzes the initial reaction of phenylpropanoid metabolism to form trans-cinnamic acid which is a precursor of lignins, flavanoids, and coumarins (Heldt & Piechulla, 2011). PPO is a Cu-containing enzyme which is also referred to as catechol oxidase, tyrosinase, phenolase, catecholase, diphenol oxidase, or *o*-diphenolase and is involved in browning development in fruits and vegetables (Jiang et al., 2016; Papoutsis & Edelenbos, 2021). Specifically, PPO is involved in the hydroxylation of monophenols to o-diphenols and oxidation of o-diphenols to o-quinones which can be converted into brown pigments (i.e., melanin) by condensation (Papoutsis & Edelenbos, 2021). Peroxidase (POD) is another enzyme involved in fruit and vegetable browning, however, it seems not to have an important role in the superficial scald development. Laccase is an ethylene dependent enzyme that was recently connected to superficial scald development in apples. Compared to PPO, laccase has a wider substrate range than PPO, since it shows both cresolase and catecholase activities (Yihui et al., 2018). Yihui et al. (2018) noted that laccase is the main responsible enzyme for superficial scald development in apples by reacting with epi-catechin to form the browning color in apple peel. The authors also suggested that one of DPA mode of action might be the inhibition of laccase activity. Superficial scald has a complicated development mechanism that needs to be further elucidated. Further research is required to investigate the involvement of PPOs and laccase in the superficial scald development since it is unclear which of the two enzymes has greater contribution.

## 4 Postharvest treatments on superficial scald reduction

### 4.1 Natural compounds

**Fig. 3:**
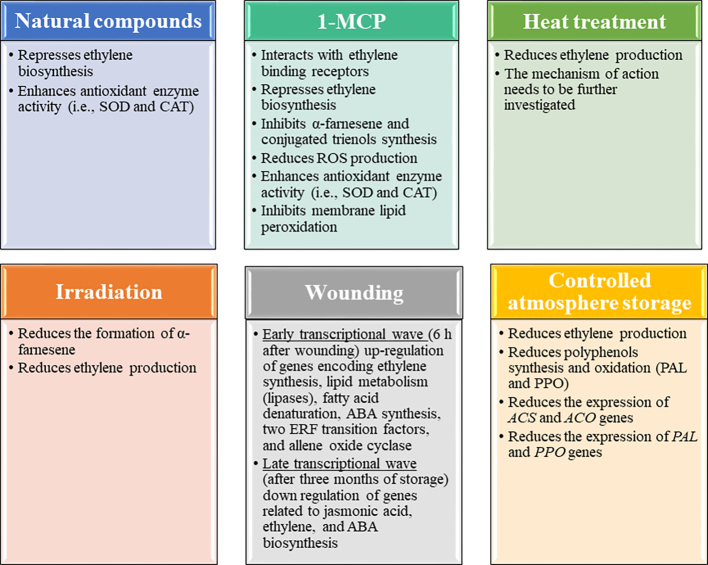
Summary of the modes of action of different treatments.

Studies have noted that natural compounds can be used as alternatives to maintain the postharvest quality of fruits and vegetables by controlling the development of pathogens and physiological disorders (Papoutsis, 2023; Papoutsis & Edelenbos, 2021). Natural compounds can be applied by spraying or dipping. The type and source of natural compounds have a significant impact on the efficiency of the treatment to reduce superficial scald incidence (Table 2). Various natural compounds (resveratrol, oregano extract, melatonin, and aloe extract) have been tested as potential treatments for the reduction of superficial scald incidence of different apple varieties (i.e., ‘Starking’, ‘Cortland’, ‘Fuji’, and ‘Starkrimson’) during cold storage (Table 2). The modes of action of natural compounds are summarized in Fig. 3. Niu et al. (2018) investigated the effects of resveratrol on ‘Starkrimson’ apple postharvest storage quality and superficial scald development over 210 days of storage at 0 °C and relative humidity (RH) 80–90% followed by 10 days of storage at 20 °C and RH of 80–90%. The authors noted that apples treated with resveratrol had lower superficial scald incidence and superficial scald index than the control which was similar to the apples treated with 1-methylcylopropene (1-MCP) and DPA. Onik et al. (2021) noted that the application of 1 mmol·L^−1^ melatonin by spraying maintained color and reduced weight loss of ‘Fuji’ apples during 56 days of storage at 1 °C and 90% RH. Melatonin maintained fresh produce postharvest quality by repressing ethylene biosynthesis and enhancing the activity of antioxidant enzymes (i.e., superoxide dismutase (SOD) and catalase (CAT)) that scavenge ROS. Natural compounds may also have a negative impact on the postharvest quality of apples by enhancing superficial scald development during cold storage. For instance, Liu et al. (2021) noted that *Aloe vera* extracts enhanced superficial scald in ‘Starking’ apples during 16 weeks of cold storage (5 °C) followed by up to 6 days of storage at 20 °C. *A. vera* extract had a negative impact on the antioxidant system of apples by reducing the activities of CAT, POD, and SOD, increasing the content of α-farnesene and its oxidation products, and by increasing the expression of *MdACS1* an important gene involved in ethylene biosynthesis. More studies are encouraged to investigate the effects of different natural compounds on superficial scald in apples and optimize the application method and conditions.

**Table 2:** Summary of studies investigating the impact of various methods on the reduction of superficial scald incidence in different apple varieties.

**Apple variety**	**Type of treatment**	**Treatments conditions**	**Storage conditions**	**Highlights**	**References**
‘Starkrimson’ apples	Natural compounds (resveratrol)	50 mg · L^−1^ resveratrol for 2 min at 20 °C	210 d at 0 °C and RH 80–90%, followed by 10 d at 20 °C and RH of 80–90%	• The treatment maintained firmness and SSC, while it inhibited α-farnesene and conjugated trienes synthesis. • Superficial scald index was 0 and 24.6%, for apples treated with resveratrol and control, respectively. • After 10 d shelf-life, the superficial scald index was 4.9 and 68.3% in apples treated with resveratrol and control, respectively. • Superficial scald incidence was 0 and 18.6% for apples treated with resveratrol and control, respectively. • After 10 d shelf-life, the superficial scald incidence was 9.1 and 83.4% in apples treated with resveratrol and control, respectively. • The PPO activity, the MDA content, and relative membrane permeability were lower in apples treated with resveratrol compared to control.	(Niu et al., 2018)
‘Fuji’ apples	Natural compounds (melatonin)	1 mmol·L^−1^ melatonin by spraying	56 d at 1 °C and 90% RH	• Melatonin treatment significantly increased POD, SOD, and CAT activities in the treated fruit. • Melatonin treatment repressed ethylene biosynthesis by down regulating the expression of *MdACS1* and *MdACO1* genes. • Melatonin treatment increased the internal melatonin level in apple fruit.	(Onik et al., 2021)
‘Cortland’ apples	Natural compounds (oregano extract and chitosan)	Oregano extract: 2 and 4 g·L^−1^ for 1 min Chitosan: 2 and 4 g·L^−1^ for 1 min	105 d at 4 °C	• Oregano extract at 2 g·L^−1^ reduced superficial scald development and conjugated trienes in apples. • No accurate conclusions could be made regarding the connection between antioxidant enzyme activities, phenolic biosynthesis, and superficial scald prevention.	(Sarkar et al., 2018)
‘Starking’ apples	Natural compounds (*Aloe vera* extracts)	Extract was evenly applied on apple surface under ambient temperature	5 °C for up to 112 d followed by up to 6 d of storage at 20 °C	• The treatment did not inhibit superficial scald development. • The treatment increased oxidative stress (ROS synthesis) as shown by the increased levels of MDA, H_2_O_2_, and superoxide anion production rates. • The treatment increased the α-farnesene and conjugated triene contents of the apples.	(Liu et al., 2021)
‘Granny Smith’ apples	Prestorage temperature combined with ultra-low oxygen	Partial pressure of oxygen of 0.2–0.5 kPa and a CO_2_ partial pressure of <0.5 kPa for 30 d at 3 °C, or 10 d at 20 °C	Up to 150 d at 0 °C, followed by post storage at 20 °C for 8 d	• Synergistic effects between storage temperatures and ultra-low oxygen treatments were found. • Acombination between temperature conditioning and ultra-low oxygen for 30 d at 3 °C had the best control of superficial scald. • Ultra-low oxygen treatment at 20 °C induces ethanol and acetaldehyde. • Ultra-low oxygen treatment reduced ethylene production and conjugated trienol concentration.	(Zoffoli et al., 2018)
‘Granny Smith’ apples	Heat treatment	Warm air (38 °C for 72 h) and hot air (42 °C for 24 h) treatments / Hot water treatment was performed by dipping apples in a water bath at 48 °C for 3 min	At 0.5 °C for up to 180 d followed by 7 d of storage at 20 °C	• Heat treatment was applied after up to 120 d of CA storage (kPa O_2_; 0.5 kPa CO_2_) at 0.5 °C followed by post-treatment air storage at 0.5 °C for up to 210 d. • Heat treatment significantly reduced superficial scald incidence in apples. • Among the different treatments, hot water was the most efficient to reduce superficial scald incidence.	(Poirier et al., 2020)
‘Granny Smith’ apples	Irradiation (x-rays)	Irradiation at 0.31 or 1 kGy	at 0–1 °C and 95% RH for up to 180 d followed by 7 d at 22 °C	• 0.31 and 1 kGy may reduce superficial scald in ‘Granny Smith’ apples through inhibition of gene expression of enzymes related to ethylene and α-farnesene biosynthesis. • Untreated samples had higher ACO activity than the treated samples.	(Martins Melo et al., 2021)
‘White Winter Pearmain’ apples	1-MCP	0.7 M 1-MCP in an airtight room (28.75 m^3^) at 22 °C for 10 h	0 °C or 5 °C for up to 113 d followed by 1 d storage at 22 °C	• 1-MCP suppressed ethylene production and inhibited scald incidence through storage of 113 d. • Storage temperature had a significant impact on superficial scald incidence. • The transcript levels of *MdHMGR1* and *MdHMGR6* in fruits stored at 5 °C were considerably higher than those stored at 0 °C. • 1-MCP resulted in the down regulation of *MdAFS* in fruit stored at 0 °C. • α-Farnesene was approximately 2-fold higher in fruits stored at 5 °C compared to those stored at 0 °C. • 1-MCP significantly inhibited the accumulation of α-farnesene and conjugated trienols.	(Ding et al., 2020)
‘Starking’ apples	1-MCP	1-MCP (1.89 g into 50 mL) was applied in fruit placed in polyethylene bags for 12 h at 20 °C	5 °C for up to 112 d followed by up to 6 d of storage at 20 °C	• The treatment maintained the content of α-farnesene and conjugated trienes in apples at a relatively low level. • The treatment effectively controlled the scald incidence and scald index. • The treatment enhanced the antioxidant system activity.	(Liu et al., 2021)
‘Cortland’ and ‘Red Delicious’ apples	1-MCP	1-MCP of 1 μL·L^−1^ in a 342 L stainless steel chamber for 24 h at 22 °C	CA storage (3.0 kPa O_2_ and 1.0 kPa CO_2_) at 0–1 °C for 210 d followed by 7 d of storage at 22 °C	• 1-MCP significantly reduced superficial scald development in both cultivars. • 1-MCP had similar results with DPA to reduce superficial scald symptoms. • 1-MCP and DPA enhanced or reduced the content of some compounds related to antiradical activities.	(Gong et al., 2021)
‘Granny Smith’ apples	Wounding	Wounding was performed with an 1 mm diameter hypodermic needle by inserting it just under the skin parallel to the fruit surface	1.7 °C for up to 90 d in CA (5% O_2_; 1.3% CO_2_; 1.7 °C;: 60/70% RH) followed by 8 d of shelf-life	• Time course RNA-Seq analyses of the transcriptional changes in wounded skins revealed two different transcriptional waves, an early one (6 h after wounding) and a late one (after 90 d of storage). • Superficial scald incidence was consistently significantly lower on the wounded half of the apple. • Wounding results in a reversed hormonal landscape for ABA, JA, and ethylene signals during later stages of post-harvest storage, which may explain the local inhibition of senescence and decay processes in wounded tissues.	(Cainelli et al., 2021)
‘Granny Smith’ apples	RLOS and DCA	RLOS + CA: RLOS (0.5% O_2_ for 10 d) followed by CA (1.5% O_2_ and 1% CO_2_ for 21 d and 0.5% O_2_ for 7 d); DCA-CF: 0.6% O_2_ and 0.8% CO_2_	Up to 300 d at 0 °C followed by 7 d shelf-life (20 °C) and 65% RH	• Harvest season has a significant impact on the efficiency of the treatments to reduce scald. • The treatments (RLOS + CA, and DCA-CF) significantly maintained the firmness and SSC of apples during postharvest storage. • The significant reduction of superficial scald during postharvest storage might be due to the suppression of the oxidation of α-farnesene and the reduced production of MHO. • The treatments maintained the antioxidant capacity and total phenolic content of the apples during postharvest storage.	(Kawhena et al., 2021)
‘Granny Smith’ apples	ULO-CA	O_2_: 0.8 kPa CO_2_: 0.6 kPa	Up to 120 d at 1 °C	• CA effectively inhibits superficial scald development in apples. • Application of 1-MCP treatment after CA can mitigate the superficial scald that develops after removing apples from CA and store them in cold air. • 1-MCP should be applied within the first week of cold storage after CA. • Scald induction resulted from cumulative oxygen exposure occurring prior to and following CA storage.	(Poirier et al., 2020)
‘Granny Smith’ and ‘Nicoter’ apples	DCA	DCA ± CF: ‘Granny Smith’ CO_2_: 1.2 kPa O_2_: 0.4 kPa ‘Nicoter’ CO_2_: 1.0 kPa O_2_: 0.55 kPa DCA ± RQ1.3: ‘Granny Smith’ CO_2_: 1.2 kPa O_2_: 0.29 kPa ‘Nicoter’ CO_2_: 1.0 kPa O_2_: 0.40 kPa DCA ± RQ1.5: ‘Granny Smith’ CO_2_: 1.2 kPa O_2_: 0.25 kPa ‘Nicoter’ CO_2_: 1.0 kPa O_2_: 0.37 kPa	Up to 270 d (1.5 °C for ‘Granny Smith’ and 3 °C for ‘Nicoter’) followed by 7 d shelf-life at 20 °C	• DCA-RQ was more efficient to control superficial scald than DCA-CF for both cultivars. • Superficial scald development was correlated to internal ethylene concentration and higher presence of oxygen in storage chamber than accumulation of α-farnesene, MHO, and 6-methyl-5-hepten-2-ol. • DCA-RQ resulted in 5 to 10-fold higher ethanol concentrations than DCA-CF. • DCA-RQ resulted in lower internal ethylene concentration than DCA-CF. • DCA-RQ resulted in higher levels of α-farnesene and MHO than DCA-CF.	(Donadel et al., 2023)

ABA: Abscisic acid.

ACO: 1-aminocyclopropane-1-carboxylic acid oxidase.

CAT: Catalase.

CA: Controlled atmosphere.

d: Days.

DCA-CF: Dynamic controlled atmosphere-chlorophyll fluorescence.

DCA+ RQ: Dynamic controlled atmosphere-respiratory quotient.

DPA: Diphenylamine.

H_2_O_2_: Hydrogen peroxide.

JA: Jasmonic acid.

1-MCP: 1-methylcylopropene.

MDA: Malondialdehyde content.

MHO: 6-methyl-5-hepten-2-one.

POD: Peroxidase.

PPO: Polyphenol oxidase.

RH: Relative humidity.

RLOS + CA: Repeated low oxygen stress + controlled atmosphere.

ROS: Reactive oxygen species.

SOD: Superoxide dismutase.

SSC: Soluble solid content.

ULO-CA: Ultra-low controlled atmosphere storage.

### 4.2 1-Methylcylopropene (1-MCP)

1-MCP is an active ingredient similar to ethylene that has been commercially applied to extend the shelf-life of various horticultural produce including apples by retarding ripening and inhibiting the development of physiological disorders (Baswal & Ramezanian, 2020; Satekge & Magwaza, 2022). 1-MCP is applied in a gaseous form after being mixed with water. 1-MCP prevents ethylene-dependent responses by interacting with ethylene-binding receptors (Baswal & Ramezanian, 2020). 1-MCP technology allows an easy application treatment that is valuable for transporting fresh produce over long distances. Even though 1-MCP is considered non-toxic from different agencies (The European Food Safety Authority (EFSA) and United States Environmental Protection Agency (USEPA)) (Baswal & Ramezanian, 2020; Boukerche et al., 2024), it has been shown that two impurities (1-chloro-2-methylpropene (1-CMP) and 3-chloro-2-methylpropene (3-CMP)) generated from 1-MCP are classified as genotoxic and carcinogenic (Boukerche et al., 2024). Boukerche et al. (2024) recently investigated the effects of 1-MCP contaminated diet on the liver function, antioxidant system, and hematopoietic parameters of albino Wistar rats for 90 days. The authors noted that 1-MCP induced oxidative stress in the liver and depleted the general enzymatic activity of the antioxidative system of the treated rats. The authors highlighted the fact that further investigation into the effects of 1-MCP when directly ingested or inhaled, targeting multiple mammalian tissues, and understanding its mode of action on a molecular level is needed (Boukerche et al., 2024). Several studies have noted that the postharvest application of 1-MCP can maintain apple quality and reduce the incidence of some physiological disorders including superficial scald (Al Shoffe et al., 2021; Gong et al., 2021) (Table 2). The efficiency of 1-MCP depends on the postharvest treatment time and storage temperature. Specifically, 1-MCP treatment needs to be applied within the first two weeks after harvest in order to effectively control superficial scald development (Poirier et al., 2020). 1-MCP treatment times and temperatures vary between 10 and 24 h and 20 to 22 °C. Ding et al. (2020) noted that 1-MCP successfully inhibited scald incidence in ‘White Winter Pearmain’ apples during 113 days of storage at different temperatures. The treatment was more efficient in apples stored at 0 °C compared to those stored at 5 °C followed by one day of storage at 22 °C. The efficiency of 1-MCP treatment can be improved when combined with controlled atmosphere (CA) storage. However, the application time of the CA storage and the treatment time of 1-MCP, when apples are removed from the CA storage are crucial parameters and they have a significant impact on superficial scaled development. For instance, Poirier et al. (2020) noted that the effectiveness of 1-MCP treatment decreased when the application was delayed by two or four weeks following removal from immediate CA storage establishment or when CA storage application was delayed by two weeks or four weeks. Several mechanisms may be involved in the effectiveness of 1-MCP application to reduce superficial scald in apples including i) reduction in the expression of ethylene synthesis-related genes (*MdACO1* and *MdACS1*) and ethylene release, ii) inhibition of α-farnesene and CTols by decreasing the expression of α-farnesene-related genes (HMGR family genes, *MdMVK*, *MdFPPS*, and *MdAFS*), iii) reduction of the production rate of reactive oxygen species (i.e., superoxide anion), iv) enhancement of antioxidant enzyme activity (i.e., SOD), and v) inhibition of membrane lipid peroxidation (Al Shoffe et al., 2021; Ding et al., 2020; Liu et al., 2021; Poirier et al., 2020). Future studies are encouraged to investigate whether 1-MCP treatment has a direct effect on the expression of α-farnesene-related genes, antioxidant system activity, and cell membrane integrity or an indirect via inhibition of ethylene synthesis.

### 4.3 Heat treatment

Heat treatment is an environmentally and human-friendly treatment that is known to maintain fresh fruit and vegetable postharvest quality by controlling the development of pathogens (i.e., fungi, bacteria) and physiological disorders (i.e., chilling injury, browning, etc.) (Papoutsis, 2023; Papoutsis & Edelenbos, 2021). Heat treatment can be applied by spraying, hot air, dipping, or steam (plant sauna) (Papoutsis, 2023; Papoutsis & Edelenbos, 2021). Optimal heat treatment conditions including treatment temperature and treatment time differ among the different fresh produce and should be carefully selected. Heat treatment temperatures above the optimal can cause irreversible epidermal cell damage. For instance, Kabelitz et al. (2019) noted that apples treated with hot water (70 °C) for 1 min showed high levels of epidermal cell damage. The epidermal damage was attributed to the deformation and destruction of cell connections. Studies have shown that the efficiency of heat treatment to reduce superficial scald in apples depends on the harvesting time. In apples heat treatment has been applied by hot air or dipping. The application of heat treatment by dipping has been proved to be more efficient to reduce superficial scald in apples comparing to hot air (Table 2). Specifically, Poirier et al. (2020) noted that dipping apples (‘Granny Smith’) in a water bath (48 °C) for 3 min immediately after harvest significantly reduces superficial scald incidence during 6 months of storage at 0.5 °C. Heat treatment can be combined with CA storage to reduce superficial scald incidence during subsequent air storage (Poirier et al., 2020). The mechanism of heat treatment to control superficial scald development in apples needs to be investigated. Although it has been noted that heat treatment significantly reduces ethylene production in apples, the efficiency of the treatment varies among the different apple cultivars. For instance, heat treatment (hot air at 38 °C for 4 days) significantly reduced ethylene production in ‘Gala’ and ‘Golden Delicious’ apples, while it had no effects on ethylene production in ‘Red Fuji’ apples (Shao et al., 2007). Therefore, it can be hypothesized that one possible mechanism of heat treatment might be the reduction of the activities of enzymes involved in α-farnesene synthesis and polyphenol oxidation via the reduction of ethylene production. Another potential mechanism might be the induction of heat shock proteins that can lead to reduced chilling sensitivity (Paull & Chen, 2000).

### 4.4 Irradiation

X-rays are ionizing irradiation that has been used in postharvest to decontaminate the surfaces of fruits and vegetables as a phytosanitary treatment, as well as to prevent quality changes caused by the development of physiological disorders (Martins Melo et al., 2021; Papoutsis et al., 2019; Yoon et al., 2020). In apples postharvest X-rays (0.31–1 kGy) treatment can effectively reduce superficial scald development during cold storage, however, the irradiation dose should be carefully selected, since non-optimal irradiation doses can cause internal quality deterioration (Table 2). Specifically, Martins Melo et al. (2021) investigated the effects of two different X-ray irradiation doses (0.31 and 1 kGy) on superficial scald development in ‘Granny Smith’ apples. The authors noted that both irradiation doses significantly controlled superficial scald incidence after cold storage for 90 days followed by storage for a week at room temperature. However, after a storage period of 180 days at 0–1 °C followed by storage at room temperature for 7 days, 72% of apples treated with 0.31 kGy showed superficial scald symptoms, with a severity index of 23.6, while no scald incidence was noted in apples irradiated with 1 kGy. However, 1 kGy induced internal browning in the treated apples after 90 and 180 days of cold storage. The authors attributed the efficiency of X-ray treatment to the reduction in the formation of α-farnesene via the suppression of the expression of *MdAFS1* that encodes α-farnesene synthase, and the reduction in ethylene production by suppressing *ACS1* gene expression. The main X-ray shortcoming is related to consumer acceptance of irradiated products (Papoutsis et al., 2019). X-ray application doses vary among countries. According to the U.S. Food and Drug Administration (FDA), the maximum X-ray irradiation dose for treating fresh fruits and vegetables to delay the maturation and ripening and inhibit the growth of decay organisms must be up to 1 kGy. The irradiated fresh food should also be labelled with a radura symbol and a text saying “treated by radiation” or “treated with radiation” (Prakash et al., 2019). More studies are encouraged to investigate the effects of ionizing and non-ionizing irradiation on superficial scald incidence and determine the optimal treatment conditions.

### 4.5 Wounding

Mechanical damage is one of the main causes of postharvest losses of fruits and vegetables. Wounds can be vulnerable points that may lead to pathogen infections (i.e., fungi, bacteria) (Al-Dairi et al., 2022; Papoutsis et al., 2019) or can enhance the synthesis of secondary metabolites that lead to the development of physiological disorders that have a negative impact on fresh produce quality (Papoutsis & Edelenbos, 2021). Membrane integrity loss is an early symptom of plant cell injuries. Two enzymes named phospholipase D (PLD) and lipoxygenase (LOX) play important roles in phospholipid catabolism, initiating lipolytic cascade in membrane deterioration in response to wounding stress (Li et al., 2017; Zhao et al., 2010). However, when wounding occurs, a series of responses are elicited in fruits and vegetables as a protective mechanism. Adenosine triphosphate (ATP) accumulation at the injury point plays a key role as the primary signal triggering ROS production (Jacobo-Velázquez et al., 2011). ROS act as a secondary signal after wounding regulating the biosynthesis of ethylene, jasmonic acid, and phenylpropanoid metabolism that results in the synthesis of phenolics, suberin, and lignin (Cisneros-Zevallos, 2018; Heldt & Piechulla, 2011; Torres-Contreras et al., 2023). Wounding has been reported to reduce superficial scald incidence in apples (Table 2). Specifically, Cainelli et al. (2021) investigated the effect of wounding on superficial scald incidence in ‘Granny Smith’ apples during three months of cold CA storage (1.7 °C, 5% O_2_, 1.3% CO_2_, and 60/70% RH) followed by 8 days shelf-life storage. The authors noted the occurrence of an early transcriptional wave (6 h after wounding) that included the up-regulation of genes encoding ethylene synthesis, lipid metabolism (lipases), fatty acid denaturation, abscisic acid (ABA) synthesis, two ethylene response factor (ERF) transition factors, and allene oxide cyclase a key enzyme involved in jasmonic acid biosynthesis. Interestingly the authors noted a late transcriptional wave of downregulation of genes involved in ABA, ethylene, and jasmonic acid biosynthesis after three months of cold storage. Given that wound healing step (i.e., temperature and duration) can significantly affect the efficiency of the treatment (Janisiewicz et al., 2016; Wang et al., 2020), future studies are encouraged to investigate the impact of different wound healing conditions on superficial scald development in apples and explore any potential connection and synergy among ethylene, jasmonic acid, and ABA.

### 4.6 Control atmosphere (CA) storage and related technologies

Gas atmosphere composition in the storage room has a significant impact on apple physiology (i.e., respiration, ethylene production, and enzyme activity) and commercial quality. The presence of O_2_ and ethylene in storage rooms may accelerate ripening and the development of physiological disorders (Al Shoffe et al., 2021; Papoutsis, 2023; Populin et al., 2023). Static CA, dynamic controlled atmosphere (DCA), ultra-low oxygen (ULO) CA, and repeated low oxygen stress (RLOS) are some of the technologies that have been developed as alternatives to DPA and ethoxyquin to control superficial scald development in apples (Table 2). CA is commercially used as an alternative to DPA and ethoxyquin for maintaining apple postharvest quality. In CA storage, air composition (20–21% O_2_, 0.03% CO_2_, 78–79% N_2_, and trace quantities of other gases) is modified by lowering O_2_ and increasing CO_2_ levels (Eskin & Hoehn, 2013). CA storage reduces or eliminates superficial scald while apples remain in storage. However, symptoms often develop after produce removal from CA (Poirier et al., 2020). The post CA storage superficial scald development can be mitigated when 1-MCP or heat treatment is applied after CA. The efficiency of the post CA storage treatments depends on the rapid establishment and maintenance of CA storage conditions and instant post-storage treatment after removal from CA storage. Poirier et al. (2020) noted that 1-MCP (1 μL·L^−1^ for 12 h) or hot water (48 °C for 3 min) treatments applied after 4 months of ULO CA storage (1 °C, 0.8 kPa O_2_; 0.6 kPa CO_2_) on ‘Granny Smith’ apples resulted in significant superficial scald incidence reduction after 4 months of subsequent air storage (0–2.5 °C) (scald incidence of 31 to 41%, 4–9%, and 2% for controls, hot water treatment, and 1-MCP treatment, respectively). It is important to note that 1-MCP application is required within the first week of cold air storage since after this period the treatment is not effective (Poirier et al., 2020). Recently the development of DCA has attracted researchers' attention. DCA can be defined as a storage system where oxygen levels are adapted from the beginning until the end of storage. In DCA, gas concentrations are monitored by sensors such as chlorophyll fluorescence (CF), respiration quotient (RQ), and ethanol (ET) (Mditshwa et al., 2018). Mainly CF and RQ sensors have been used in studies aiming to maintain apple quality by reducing superficial scald development (Table 2). Lower oxygen limit (LOL) is defined as the environmental O_2_ level where cell metabolism changes from aerobic to fermentative. During the storage of fruits and vegetables, the O_2_ levels should be set above LOL (Wright et al., 2012). Both CF and RQ detect LOL in different modes. For instance, CF sensors detect O_2_ level changes depending on physiological response. This is achieved through the non-destructive monitoring of the minimum CF parameter (F_o_) which is sensitive to low-O_2_ stress using sensors (Mditshwa et al., 2018; Wright et al., 2012). RQ sensors monitor directly LOL based on the ratio between CO_2_ production and O_2_ uptake (Thewes et al., 2017). DCA-CF is more widely used than DCA-RQ in the commercial storage of apples (Donadel et al., 2023). Between DCA-CF and DCA-RQ, the latter has been reported to be more efficient to reduce superficial scald incidence in apples, however, DCA-RQ may result in significantly higher ethanol concentrations which is an index of anaerobic metabolism. Donadel et al. (2023) investigated the effects of DCA-CF and DCA-RQ on superficial scald incidence in ‘Granny Smith’ and ‘Nicoter’ apples during up to 9 months of storage (Table 2). The authors noted that even though DCA-RQ was more efficient to control superficial scald than DCA-CF for both cultivars, DCA-RQ resulted in 5 to 10-fold higher ethanol concentrations than DCA-CF, which is an indication of anaerobic metabolism. This could be due to the high sensitivity of CF sensors to O_2_ stress (Mditshwa et al., 2018). The authors noted that superficial scald development was more correlated to the internal ethylene concentration and higher presence of oxygen in the storage chamber than the accumulation of α-farnesene, MHO, and 6-methyl-5-hepten-2-ol. Transcriptomic studies have shown that during ULO and DCA storage the expression of genes related to ethylene production (*ACS* and *ACO*), polyphenols synthesis and oxidation (*PAL* and *PPO*) are reduced (Populin et al., 2023). During DCA and ULO storage, the transition from aerobic respiration towards an anaerobic metabolism (fermentation) in apples is driven by two classes of genes belonging to *pyruvate decarboxylase* and *alcohol dehydrogenase* gene families (Hormazabal-Abarza et al., 2024; Ventura et al., 2020). Pyruvate decarboxylase is responsible for the non-oxidative decarboxylation of pyruvate into acetaldehyde which is subsequently converted into ethanol through alcohol dehydrogenase activity (Hormazabal-Abarza et al., 2024). This process is established by the plant in order to produce energy in the scenario of oxygen deprivation ensuring physiological activity (Populin et al., 2023). To sum up, the efficiency of DCA or CA to control superficial scald development in apples is affected by several parameters including variety, storage temperature, storage time, harvesting season, gas concentration, sensor type, and maturity stage at harvest (Donadel et al., 2023; Kawhena et al., 2021; Mditshwa et al., 2018; Populin et al., 2023; Wright et al., 2012). More research is required to investigate the impact of both CF and RQ sensors during DCA storage on apple physiology and superficial scald development.

## 5 Future directions

Recent studies have made a significant contribution to the understanding of superficial scald development in apples during cold storage. However, there is still a need to investigate potential synergies among ROS, phenolics, α-farnesene, and plant hormones in the development of superficial scald. To date, studies usually investigate superficial scald development on one apple cultivar. The use of more than one apple cultivar in future research will help to identify specific genes that encode metabolites that are involved in apple resistance to superficial scald. Future studies are encouraged to investigate alternatives to DPA and ethoxyquin considering the safety of the method for human health and the environment. The development of commercial postharvest treatments could be achieved by conducting large-scale experiments and through the collaboration between academia and industry. More research is also needed to optimize the DCA and ULO conditions to mitigate any negative impacts of ultralow oxygen concentrations. Finally, more toxicological studies are required to provide information on DPA and ethoxyquin toxicity on human and animal health and the environment.

## CRediT authorship contribution statement

**Konstantinos Papoutsis:** Writing – review & editing, Methodology, Investigation, Conceptualization.

## Declaration of competing interest

The author declares that there is no conflict of interest.

## Data Availability

No data was used for the research described in the article.
